# Optimizing and Evaluating the Transdermal Permeation of Hydrocortisone Transfersomes Formulation Based on Digital Analysis of the *In Vitro* Drug Release and *Ex Vivo* Studies

**DOI:** 10.2174/2667387816666220608115605

**Published:** 2022-10-19

**Authors:** Asmae Abdelwahd, Bazigha K. Abdul Rasool

**Affiliations:** 1 Pharmaceutics Department, Dubai Pharmacy College for Girls, Muhaisnah-1, Dubai, United Arab Emirates

**Keywords:** Transfersomes, hydrocortisone, DDSolver, *in vitro* release, transdermal, permeation, edge activator

## Abstract

***Background*:** Transfersomes can be used to enhance transdermal drug delivery due to their flexibility and ability to incorporate various molecules. For example, hydrocortisone (HC), a corticosteroid, is taken by different routes and serves as immunosuppressive, anticancer, and antiallergenic; however, it is poorly absorbed by the skin.

***Objective*:** Therefore, the current study suggested HC-loaded transfersomes as an alternative route of administration for reaching deeper skin layers or systemic circulation, to reduce the side effects of HC and improve its bioavailability.

***Methods*:** HC transfersomes were prepared by the thin-film hydration method and characterized for their vesicular size, zeta potential, drug entrapment efficiency, elasticity, FTIR spectroscopy, *in vitro* drug release, *ex vivo* permeation, and irritancy in rabbits. The optimized formulation, F15 (containing HC 20 mg, egg phosphatidylcholine (EPC) 400 mg, and 75 mg of Span 80), was chosen because it showed the highest (*p*< 0.05) EE% (60.4±0.80) and optimized sustained *in vitro* drug release (Q8 = 87.9±0.6%).

***Results*:** Extensive analysis of the drug release data from all formulas was performed using the DDSolver software which quantitatively confirmed the successful formulation. The Weibull equation was the best model to fit the release data compared to others, and the release mechanism was Fickian diffusion.

***Conclusion*:** The simulated pharmacokinetic parameters showed that F15 had the highest AUC, MDT, and DE. Furthermore, F15 significantly enhanced HC permeation by 12-folds compared to the control through the excised rat's skin. The skin irritancy test has proven F15 safety and skin compatibility.

## 1 INTRODUCTION

Although the skin is a barrier to harmful substances, it is used for topical and transdermal drug delivery. Transdermal drug delivery can protect the human body against side effects caused if the drug is given orally or *via* other routes. Also, it improves the drug's bioavailability, prolongs the duration of action, and offers self-administration, thus improving the patient compliance with the medication [1]. However, not all drugs are justified for transdermal therapy due to their low cutaneous permeability. Skin structure, specifically the stratum corneum layer, is the main absorption barrier. The stratum corneum, the outermost layer of the skin, is lipophilic and composed of dead corneocyte cells filled with cross-linked keratins and lipids. Transport across this layer is mainly through diffusion, so lipophilic drugs are more permeable to the skin [2].

The application of nano/microtechnology for transdermal dosage forms is the future mean of therapeutic delivery [3]. Unfortunately, some of these systems are still under clinical trial. Indeed, nano/microstructures are an expansive definition of materials engineered on nano/micro-scale, the most commonly used ones for transdermal delivery include nano/microvesicles, which might be lipid-based carriers such as liposomes, niosomes, and ethosomes [4, 5]. These systems have shown high success in breaching the skin and reaching the dermis for systemic circulation. However, the stratum corneum remains a barrier to the above-mentioned vesicular systems for efficient skin absorption. On the other hand, the deformable vesicle transfersomes seem to be capable of overcoming many obstacles.

Moreover, transfersome is an elastic liposomal carrier described as a deformable lipid-based droplet. It permits easy penetration through pores that are much smaller than their size. They can pass through narrow constrictions from 5-10 μm, which is less than their diameter, without substantial loss. The structure of the transfersome vesicles is complex, highly adaptable, and stress-responsive. Transfersomes comprise phospholipids and edge activators (EAs) like sodium cholate, Tween 20, Span 80, and dipotassium glycyrrhizinate phospholipids. The edge activator is typically a single-chain surfactant that imparts instability to the lipid bilayer in the vesicle and enhances the elasticity or fluidity of the vesicle by decreasing its interfacial tension. Fortunately, transfersomes can deliver drug molecules with high efficiency either into or *via* the skin, depending on the administration route or application choice. In addition, transfersomes are biodegradable, biocompatible, and resistant to metabolic degradation [6].

HC is the pharmaceutical form of cortisol, a glucocorticoid hormone generated at a rate of 12 mg to 30 mg per day in a healthy body [7]. It can be used in many conditions, like orally for inflammation and in adrenal replacement therapy. Dermal dosage forms include ointments, microemulsions, and aqueous gels [8]. In addition, many conditions are treated with dermal hydrocortisone, including atopic dermatitis, vitiligo, eczema, and alopecia areata [9].

The oral half-life of HC is 1.8 hours and is almost equal to that of an intravenous injection (1.7 hours) [10]. In addition, topical HC cream showed limited permeation into the deep layers of the skin, where topical bioavailability is 4-19% and T_max_ is equal to 24 hours [11]. In previous studies, researchers have attempted to enhance the absorption of HC across the skin by loading the drug with vesicular carriers. For example, liposomes loaded with HC were prepared and showed improved concentration-time profiles and higher HC concentration in the dermis [12]. However, transfersomes of interest to us have hardly been mentioned in the literature on corticosteroids, especially HC [13, 14].

Accordingly, we prepared and characterized different HC transfersome formulations using EAs with different hydrophilic-lipophilic balances (HLB). DDSolver software was used to analyze the *in vitro* release data to determine the kinetic modeling and mechanism of drug release. It also helped in the selection of the optimized formulation, which was subjected to further *in vitro* evaluation and skin permeation studies. FTIR spectroscopy analysis was also performed to investigate the possibility of drug-excipient interaction.

## 2 MATERIALS AND METHODS

### 2.1 Materials

HC pure powder was a kind gift from Julphar, Ras Al Khaimah, UAE. Egg phosphatidylcholine was purchased from Sigma, China. Polyoxyethylene (20) cetyl ether (Brij 58^®^), and polyoxyethylene(20) cetyl ether (Brij 52^®^) were obtained from Aldrich Chemical Company, USA. Sorbitan monolaurate (Span 20^®^), sorbitan monooleate (Span 80^®^), and sodium cholate were purchased from (Aldrich, Germany). All other used chemicals and solvents were of analytical grade. The reference product, hydrocortisone lotion 1%, was purchased from Mericon Industries, Inc.

### 2.2 Preparation of HC-loaded Transfersomes

HC-loaded transfersomes were prepared by the thin-film hydration method, as shown in Fig. (**1**). First, HC (20 mg) and edge activator were added to a phospholipid EPC (400 mg), and all components were dissolved in 12 mL of absolute ethanol. Then, the mixture was sonicated for 5 minutes in a sonicator (S60H, Elmasonic Germany) at room temperature to ensure complete dissolution. The solvent was then evaporated under vacuum in a rotary evaporator (Laborota 4000 efficient, Heidolph) at 60 ^o^C, and 250rpm. A thin lipid film was formed. The film was hydrated with 9 mL of PBS pH 7.4 for one hour. To obtain uniform nanosized vesicles, the samples were sonicated for 10 minutes and extruded 15 times through a Millipore^®^ filter (pore size equal to 0.22 μm). The samples were placed in well-closed plastic containers, wrapped with a parafilm, and stored in a cold, dry, and dark place. The compositions of HC transfersome formulations are shown in Table **1**.

The lyophilized samples were prepared by placing the transfersomal dispersion in wide-mouth glass vials. They subsequently were frozen at -65.4 °C and pressure of 7x10^-2^ mbar for 24 h by a Biobase freeze drier (Bk-FD 10P, China). Then, the vials were tightly closed, wrapped with a para-film, and stored.

### 2.3 Preparation of HC Calibration Curve

The calibration curve of HC was prepared by making different dilutions (1, 2, 3, 4, 5, 6, 7, 8, 9, and 10 μg/mL) from a stock solution of 0.1% HC in PBS pH 7.4. The absorbance of the prepared aliquots was measured using a UV-visible spectrophotometer (UV1700, Shimadzu, Japan) at λ_max_ 246 nm against the blank solution (PBS pH 7.4). The λ_max_ was determined from the UV spectrum at the scanning range of 200-400 nm. The calibration curve was obtained by plotting the concentration (μg/mL) *versus* absorbance in a Microsoft^®^ Excel 2020 spreadsheet. The linear equation and the correlation coefficient (R^2^) were generated and as follows: y = 0.0402x + 0.0067; R^2^ = 0.9997. The high value of R^2^ indicated good compliance with Beer-Lambert Law.

### 2.4 Morphological Examination

The transfersomes were detected, and then photos were captured under an optical microscope fitted with a digital camera (Carl Zeiss, Germany) at 40X magnification. This test was performed before particle size reduction by ultrasonication and extrusion.

Furthermore, the surface topography of the selected F15 and the blank formula was examined by Thermo Scientific Apreo Scanning Electron Microscope SEM (Czech Republic).

The lyophilized samples were placed on an aluminum holder using carbon tape, followed by a gold coating. Afterward, the samples were subjected to SEM imaging.

### 2.5 Vesicle Size, PDI, and Zeta Potential Measurements

The vesicle size, Poly Dispersibility Index (PDI), and zeta potential were determined using a Zetasizer (Anton Paar, Litesizer 500, Germany). The cell was cleaned with distilled water and ethanol before placing the sample. First, the sample was prepared by diluting 0.1 mL of the transfersomes with 10 mL of PBS pH 7.4. Then 1 mL was collected from the sample placed in the cell and made sure that it was free from bubbles before starting the measurement. The results were recorded as mean ±SD (n=3).

### 2.6 Elasticity Measurement

The transfersomal formulations were extruded at 7.5 psi pressure while subjected to polycarbonate filter membranes (Sigma Chemicals, UK) of pore size 0.22 µm. A sample (0.5 mL) of the transfersomal formulation was diluted up to 10 mL with PBS pH 7.4, extruded for 5min through the membrane, weighed, and the mean of vesicle size was measured by the Zetasizer at room temperature [15]. The transfersomes elasticity (E) was calculated using the equation below:

E = Jflux [ RV/RP]^2^ Eq. (1)

Where: Jflux: the rate of penetration through a permeability barrier [mg·sec^-1^·cm^-2^], RV: the size of vesicles after extrusion, RP: the pore size of the filter medium.

### 2.7 *In Vitro* Drug Release Study

The *in vitro* release studies of the drug-loaded transfersomes were performed using the vertical Franz diffusion cell (Copley Scientific Limited, UK). A cellophane membrane (Millipore HVLP, pore size 0.45 µm) was placed on the diffusion cell. The receptor compartment was filled with 22.5 mL of PBS pH 7.4. The fluid was thermostated at 32± 0.5 ^o^C and agitated at 100 rpm. The transfersomal dispersion (0.3 mL) was placed in the donor compartment. Samples of 2mL were withdrawn at suitable time intervals (0.5, 1, 1.5, 2, 3, 4, 5, 6, 7, and 8 h) and replaced with an equal volume of PBS pH 7.4. This step is essential to preserve the sink condition. The collected samples were analyzed for the content of the diffused drug by a UV-VIS spectrophotometer (UV1700, Shimadzu, Japan) at λ_max_ 246 nm. The concentration of drug released was then computed from the linear regression equation (y = 0.0402x + 0.0067) generated from the previously prepared calibration curve.

### 2.8 Drug Release Kinetics Modeling Using DDSolver Software

#### 2.8.1 Kinetic Modeling of Drug Release

In Microsoft Excel, DDSolver was employed to assess the drug release kinetics and compared data to a reference product [16]. The derived kinetic models include Zero-order, First-order, Higuchi, Hixson-Crowell, Korsmeyer-Peppas, Hopfenberg, Baker-Lonsdale, and Weibull (Table **2**). The correlation coefficient R^2^ and rate constant (k) were obtained. In addition, the release exponent “n” in the Korsmeyer-Peppas model was identified to deduce the mechanism of release from the transfersomal formulations. Finally, the R^2^ adjusted and other statistical criteria, Akaike Information Criterion (AIC) and Model Selection Criterion (MSC) were computed.

#### 2.8.2 Visual Goodness of Fitting

Further analysis to confirm the selected kinetic model was performed. Visual Goodness of Fitting (GOF) evaluation was performed through the residuals (Qo-Qc) correlation *versus* time and the released amount of drug against the time of the observed data (Qo) compared to the predicted values (Qc) for the selected modeling.

#### 2.8.3 Simulated Pharmacokinetic Parameters

The simulated pharmacokinetic parameters, including area under the curve (AUC), mean dissolution time (MDT), and dissolution efficiency (DE), were obtained from the DDSolver based on the entered *in vitro* release data of the formulas compared to the reference product.

### 2.9 FTIR Spectroscopy Study

FTIR analysis was performed using a JASCO FT/IR-6300 FTIR spectrometer (Germany). Five milligrams of each sample were mixed with 30mg of KBr and compressed at 10 tons by a hydraulic press. The spectrum was recorded for the pure drug (HC), EPC, and F15. The scanning range was 400 cm^-1^ - 4000 cm^-1^ with a 2 cm^-1^ resolution.

### 2.10 *Ex Vivo* Permeation Study

#### 2.10.1 Skin Preparation

The *ex vivo* permeation study was performed in the Pharmacology Department of Dubai Pharmacy College For Girls. Female albino rats (body weight, 250-300 g) were sacrificed using excessive chloroform inhaled. The abdominal skin was chosen to perform this study. First, the skin’s hair was removed using shaving cream and defatted by whipping with a piece of cotton soaked with diethyl ether. Next, the skin was cut into square pieces (3 cm^2^). Finally, PBS was used to wash the skin pieces, kept in the freezer at –20 ^o^C, and warmed to room temperature before use [17]. The Animals' Ethical Committee, Research Unit-Dubai Pharmacy College for Girls, approved this study (Reference No. REC/MPharm/PPD/2019/02RE).

#### 2.10.2 Experimental Design

The *ex vivo* permeation studies of HC-loaded transfersomes, compared to the reference product and control (HC suspension in PBS, pH 7.4), were conducted using Franz-diffusion vertical cells (Copley Scientific, UK). In between the donor and receptor compartment, the rat’s skin was placed where the stratum corneum and the dermis were in direct contact with the sample and the receptor compartment, respectively. The receptor compartment was filled with 22.5 mL of PBS pH 7.4 and maintained at 32± 0.5 ^o^C and 100 rpm. Samples of 2mL were withdrawn at suitable time intervals (1, 2, 3, 4, 5, 6, 7, and 8 h). Samples 2ml were withdrawn and replaced with an equal volume of diffusion medium to maintain the sink condition. UV-Vis spectrophotometer (UV1700, Shimadzu, Japan) was utilized to analyze the collected samples for the drug content with λ_max_ at 246 nm.

#### 2.10.3 Data Analysis

The results were plotted on a Microsoft^®^ 2020 Excel spreadsheet to determine the flux Jss, the slope of the linear portion of the cumulative amount of HC permeated per unit area (mg/cm^2^) against time (h). The permeability coefficient P (cm/h) was calculated as follows:


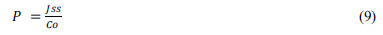


Where: Co: Initial concentration, Jss: Drug’s flux (mg/cm^2^.h^-1^); P: Drug’s permeability coefficient (cm/h).

The permeation enhancement ratio (ER) was also computed with the following equation:


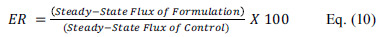


### 2.11 Skin Irritation Test

The Animals' Ethical Committee, Research Unit at Dubai Pharmacy College for Girls, approved the skin irritancy test (Reference No. REC/MPharm/PPD/2019/02). The test was performed following the ASTM method F719–81 [18]. Three female albino rabbits weighing approximately 1 kg were used in the test. The animal's dorsal side was carefully shaved, and three circular areas of approximately 50 cm^2^ were drawn on the animal's back. The restricted areas were wiped with a formaldehyde aqueous solution (20%), and then the solution was allowed to evaporate. Approximately 0.5 g of the formulation F15 and HC powder were placed on two circular areas. Known irritancy (histamine) control substance was injected intradermally into the third circular area for calibration. The back of the animal's ear was shaved, and a piece of cotton immersed in a small amount of xylene was applied to the shaved portion to dilate the superficial ear vein. Next, 1 mL of Trypan blue 0.5% was gradually injected into the selected vein. The irritation level was assessed depending on the accumulation of Trypan blue in the treated area. The degree of blueness was visually ranked to provide a relative order of irritancy of the tested substance. The observations were made after 1, 6, 12, and 24 h.

### 2.12 Statistical Analysis

All results were expressed as mean values ±SD (n=3). One Way ANOVA test was employed to determine statistically significant differences at a significance level of p< 0.05. These tests were performed using IBM SPSS^®^ statistics version 1.0.0.1508.

## 3 RESULTS AND DISCUSSION

### 3.1 Preparation of HC-loaded Transfersomes

The thin-film hydration technique used to prepare the transfersomes was successful in the initial trials. The optimized conditions for the transfersomes preparation were: solvent: ethanol (12 mL); hydration medium: PBS (9 mL); hydration time (1 h); and temperature (25 ^o^C). Moreover, our results were in agreement with those from previous studies [19].

### 3.2 Morphological Examination

The optical microscope examination was conducted to detect the existence and shape of HC transfersomes in the formulation. The vesicles were found to be intact entities and of a spherical shape. They were also abundant in the field. The microscopic images of some of the prepared transfersomal formulations are shown in Fig. (**2**).

### 3.3 Particle Size, PDI, and Zeta Potential Measurement

The vesicle size of the HC transfersomes ranged from 839 nm to 2269 nm (Table **3**). The smallest particle size was observed with a Span 80. The sonication technique was used for particle size reduction. Nevertheless, further size reduction was achieved by extruding the samples. Our results were in agreement with those of a previous study by Morsi *et al.* [20], who also produced transfersomes on a nano-micron scale.

It appears that there is a rational relationship between the hydrophilic-lipophilic balance (HLB) values of EAs and the mean particle size of the transfersomes. The lower the HLB of the surfactant, the smaller the particle size (Table **3**). The particle size of the transfersomes was in the following ranks: Span 80 < Span 20 < Brij 58 < SC. However, Brij 52 did not follow the order and produced large vesicles, even though it had an HLB value <10. The HLB values of the EAs are demonstrated in Fig. (**3**). Similar results were reported by Qushawy *et al.* [21]. They proved that lipophilic surfactants (HLB< 10), like Spans, showed the smallest particle size compared to the hydrophilic surfactants (HLB> 10), which produced a larger particle size.

At another point, the physical state of surfactants may also partially explain the size difference, where Span 80 and Span 20 have a liquid nature while SC, Brij 52, and Brij 58 are solids at 25°C. In addition, the nonionic EAs like Span 20 and Span 80 showed an increase in particle size with their numbers, which may be attributed to the volume of their polar heads [22]. The phospholipid weight ratio of EA also affected the vesicles' size for all EAs except for SC. The particle size was significantly (*p*< 0.05) increased by increasing the phospholipid's weight ratio to the EA. Apigenin, a lipophilic drug similar to HC, showed a significant correlation between the phospholipid to EA ratio and the particle size. The higher the ratio, the higher the particle size [23].

The PDI ranged between 18% and 36% (Table **3**) and were within the accepted value (< 50%), indicating the homogeneous distribution of the vesicles throughout the colloidal dispersion and a narrow deviation from the mean size [24]. Also, Duangjit and his team reported similar results [24, 25]; they prepared MX-loaded cationic transfersomes of similar particle size (0.2-0.3 μm) and considered them homogeneous with uniform particle size distribution.

The zeta potential of the prepared transfersomes was also determined. Generally, the vesicles are optimized when the zeta potential is more than ±15 mV because of lower particle aggregation due to a high repulsive force [26]. For small particles, high zeta potential means more stability [27]. Moreover, a negative charge favors the skin permeation of transfersomes [27].

The nature and magnitude of the electrical charge of the particles are influenced by the chemical nature and amount of the formulation excipients, *i.e*. surfactant and phospholipids. In the present study, the vesicles in all prepared transfersome formulations were negatively charged and their zeta potential values ranged from -3.5 mV to -10.1 mV (Table **3**). The phospholipid EPC used was the main factor responsible for the negativity of the particles. Since EPC is a zwitterionic compound (IEP = 6-7), it carries a negative charge at pH 7.4 [27]. The negative value of the zeta potential of SC-containing vesicles, was higher than that of Span 80 and Brijs, as the latter two EAs are non-ionic compounds, while SC is an anionic compound EA [27]. However, no significant difference (*p* < 0.05) was found between the zeta potential of Span 80-containing formulas and SC transfersomes at all concentrations. Furthermore, the concentration of all EAs used in the formulations affected the zeta potential value of the particles. Our results are in agreement with those of Bnyan *et al* [19], who had prepared lidocaine-loaded transfersomes using EPC and sodium deoxycholate, Tween 80, and Span 80 as EA.

### 3.4 Entrapment Efficiency

HC-loaded transfersomes showed an entrapment efficiency percentage between 16.1% and 69.4% (Table **3**). The highest EE% (p< 0.05) was obtained with Span 80, whereas the lowest EE% was seen with Brij 58. The EE% of HC in the transfersomes was EA dependent, and it was in the following order: Span 80 > Brij 52 > Span 20 > SC > Brij 58.

First, the EAs’ structure was found to affect drug loading so that a longer alkyl chain leads to higher EE%. For example, the highest drug loading was obtained with Span 80 due to its longest alkyl chain (18 C atom) compared to other EAs (Span 20: 12C; Brij 52 and Brij 58: 16C; SC: none). The same effect was observed in previous research, with higher entrapment efficiency with edge activator of longer alkyl chains, namely, sodium deoxycholate [28, 29].

The HLB of the EA is a second factor. Regarding lipophilic drugs like HC, it is expected that the lower the HLB value, the more lipophilic EA, and the higher the drug entrapment capacity. Results indicated that Span 80, Brij 52, and Span 20 have HLBs less than ten, making them more lipophilic and, therefore, showing higher EE%. In contrast, Brij 58 and SC have HLB of more than ten or more hydrophilic surfactants, causing less drug’s EE%. Similar results were obtained previously with miconazole transfersomes where the highest EE% showed by Span 80 containing formulations [21]. Indeed, Brij 58’s chemical structure possesses large polar head groups, which decreases its capacity to make spherical vesicles and consequently reduces its entrapment efficiency [30]. While Span 80 is a nonionic surfactant and has many advantageous characteristics on transfersomes. It stabilizes the phospholipid bilayer components, increasing the drug's entrapping efficiency in the vesicles and improving the transfersomes flexibility, leading to better drug penetration into the vesicles [31].

Another fact is that a high amount of EA in the formulation may lead to solubilization of the drug by micellization. Therefore, the drug’s diffusion into the aqueous phase was enhanced at the time of transfersomes’ preparation [6]. The same effect was observed with SC, Span 20, and Brij 58. The edge activator's high concentration also increased the vesicle number, favoring the entrapment efficiency in a higher volume of hydrophobic bilayer domain. The latter entrapped the hydrophobic drug, which was observed explicitly with Span 80. However, when the amount exceeds the vesicles' EE%, leakage from the disrupted membrane lowers EE% [19]. Therefore, Brij 52 presented an irregular loading efficiency characterized by an unexpected decrease in EE value which is due to the drug’s leaching.

### 3.5 Vesicles’ Elasticity

Elasticity is what differentiates transfersomes from conventional liposomes. With the help of EAs, transfersomes are more flexible and can pass through tiny pores. Besides, their elasticity lowers the chance of vesicular rupture [19]. Research on the mechanism that grants elasticity to transfersomes has revealed that they perform structural changes in the stratum corneum, which is seen with thread-like channels formed when transfersomes contact the skin, easing their permeation. In comparison, similar channels were not observed with conventional liposomes [32].

The results suggested that both EA type and amounts were significantly (*p*< 0.05) influencing transfersomes elasticity (Table **3**). The optimum amount that led to the highest elasticity for most transfersomal formulations was 75mg (16:3 weight ratio). In addition, SC showed the highest elasticity, followed by Span 80 and Span 20. While Brij 52 and Brij 58 showed the most negligible elasticity. It was noted that the higher the EA amount, the greater the elasticity. This increase might be due to lipid bilayer fluidization. Our findings were in agreement with AL Shuwaili *et al.* [15]. Indeed, SC made the transfersomal vesicles very elastic because it softens the membrane and offers more flexibility, while spans were reported to show less elasticity and lower viscosity [15].

### 3.6 *In Vitro* Release Studies

The *in vitro* release profiles of all transfersome formulae showed a burst effect during the first four hours, followed by a sustained release pattern. The time to reach steady-state was after four hours of the run, Fig. (**4a**-**e**). The burst effect could be linked to the lipophilic nature of HC molecules and their location in the vesicles' lipid bilayers; therefore, the vesicular bilayers' breakage is caused by vesicle deformation during the molecule permeation [22].

F15 showed the highest *in vitro* release (Q8) with an 87.9% drug release value compared to other formulations (Table **3**). Statistically, there was an indication of a significant difference [*p*< 0.0^5^] between *in vitro* release and different EAs. Moreover, it is reported that the higher the entrapment efficiency, the higher the *in vitro* release [33], which indicated a relationship between EE% and % release.

### 3.7 Drug Release Kinetics Modeling

The *in vitro* drug release data were fitted to different kinetic models built in the DDSolver software, including Zero-order, First-order, Higuchi, Korsmeyer-Peppas, Hopfenberg, Baker-Lonsdale, and Weibull [16, 34]. The best model would be the one with the highest R^2^ (closest to 1). The results of the release data analysis showed that all prepared formulations fitted the Weibull model as the R^2^ adjusted values were significantly (*p*< 0.05) highest than other models, indicating its suitability to describe HC release from the transfersomes (Table **4**).

Based on the shape parameter 𝛽 from the Weibull model, the drug was released according to the Fickian diffusion because 𝛽 ≤ 0.75 [34]. The “Ti” parameter is the time interval before the start of dissolution and is usually close to zero. In our findings, the Ti values were between 0 and 1 [16]. Furthermore, by analyzing the Korsmeyer-Peppas model, the value of “n” for all formulations was less than 0.5, confirming the Fickian release mechanism of HC from the transfersomes [35]. For the reference product, 𝛽 and n values were > 0.5 and < 1, indicating the anomalous transport case *i.e*., HC release occurred by diffusion and erosion [36].

The software also generated additional kinetic parameters, including AIC and MSC, that were used to confirm the proper kinetic model selection. AIC with the smallest value and MSC with the highest value refers to the best fit model. According to these parameters, the best data fitting was again to the Korsmeyer-Peppas and Weibull models (Table **4**).

Moreover, visual GOF was conducted by generating the correlation of residuals (Qo-Qc) *versus* time to confirm the best modeling selection. As shown in Fig. (**5**) (F1-F15), it was noticed that the Weibull model showed superiority over other models as its graphical representation was close to the x-axis for most formulations, indicating that the Qo (observed) and Qc (predicted) are close to each other. In addition, F15 was a very prominent candidate that exhibited the best results among the other formulations.

**Fig. (6):**
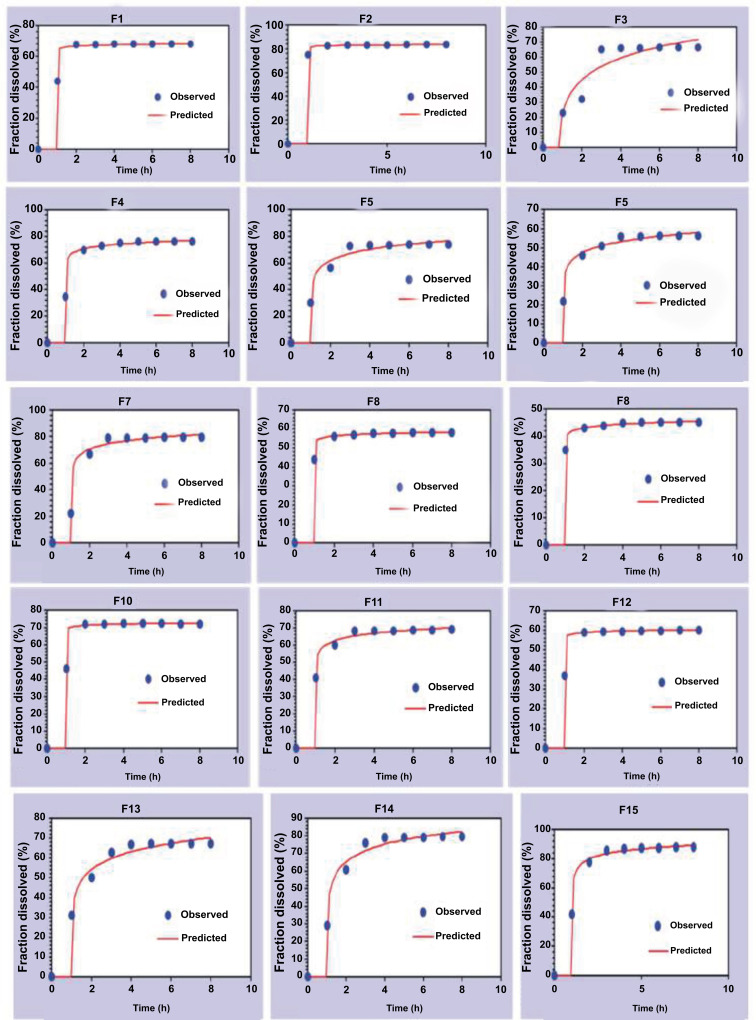
The correlation of HC released amount (Qo) *versus* the predicted amount of HC released (Qc) of F1-F15 by the Weibull model, generated by DDSolver.

Additionally, the correlation between the amount of HC released (Qo) and the amount of HC anticipated to be released (Qc) by the F1–F15 Weibull model were compared (Fig. **6**). The results confirmed the previous findings that F15 was one of the best formulations fitting the order of kinetic. However, the Weibull model is not deduced from any kinetic fundament. It is an empirical model presenting limitations, *e.g*., limited use of establishing *in vivo /in vitro* correlations [34].

Regarding the simulated pharmacokinetic parameters generated by DDSolver, a significant difference was noted amongst the formulations of different EAs. The AUC was significantly increased (*p*< 0.05) with the highest increment in candidate formula F15, proving that our drug was more bioavailable than the reference. In addition, MDT and DE showed a significant difference (*p*< 0.05) amongst different EAs, with the highest increment shown for F15 (Table **5**). Thus, the solubility and dissolution rate of HC increased due to its incorporation into the nano lipid vesicles (transfersomes). Other studies have also proposed nano/microparti- culate delivery systems to improve the solubility and dissolution rate of lipophilic hypoglycemic drugs [37].

Compared to other formulations, the EAs significantly impacted the pharmacokinetics parameters and portrayed F15, the candidate formula. F15 (containing 75mg of Span 80) primarily displayed the highest (*p*< 0.05) entrapment efficiency and *in vitro* release (Q8 = 87.9%) after eight hours of the run, compared to other formulations. Even though the results from the simulated PK parameters, AUC, MDT, and DE, proved that our selection was correct. Also, the vesicular carriers as transfersomes were portrayed as advantageous in drug delivery due to their positive impact on drugs’ bioavailability and dissolution efficiency. For example, the AUC increment was reported with timolol maleate transfersomal gel [20].

F15 had proceeded for further *in vitro* evaluation. F15 was subjected to FTIR spectroscopy analysis to study the possibility of any drug-lipid interaction and was also examined under scanning electron microscopy. Moreover, the *ex vivo* permeation of HC from F15 compared to a reference product and control was studied.

### 3.8 FTIR Spectroscopy Analysis

**Fig. (7):**
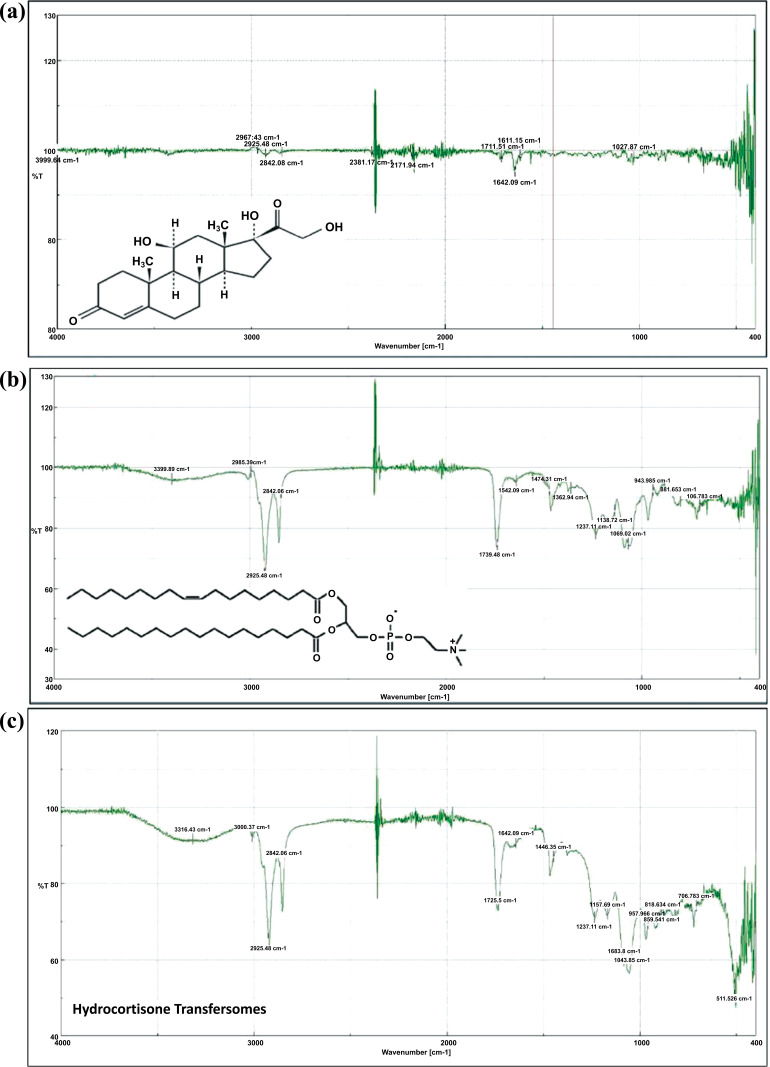
FT-IR spectra of **a**) HC; **b**) EPC; and **c**) F15, respectively.

1. HC spectrum, Fig. **7**(**a**):

o Molecular formula: C_21_H_30_O_5_.

o The prominent absorption bands of HC are 2967cm^-1^ and 2842cm^-1,^ indicating C-H bonds,

o Peaks at 1711cm^-1^ and 1642cm^-1^ indicate C=O and C=C bonds,

o Peaks at 1027cm^-1^ indicate C-C and C-O bonds.

2. EPC spectrum, Fig. **7**(**b**):

o Molecular formula: C_44_H_84_NO_8_P.

o EPC peaks include 2995cm^-1^, 2925 cm^-1^ and 2842 cm^-1^ indicating C-H,

o They also include 1739 cm^-1^ and 1642 cm^-1^ indicating C=O,

o Peaks at 1474 cm^-1^, 1362 cm^-1^, 1237 cm^-1^ and 1069 cm^-1^ indicating C-C and C-N and C-O.

3. F15 spectrum Fig. **7**(**c**):

All characteristic peaks of the drug and lipid appeared in the spectrum of F15, indicating no drug-excipient interactions.

### 3.9 Scanning Electron Microscopy (SEM)

**Fig. (8):**
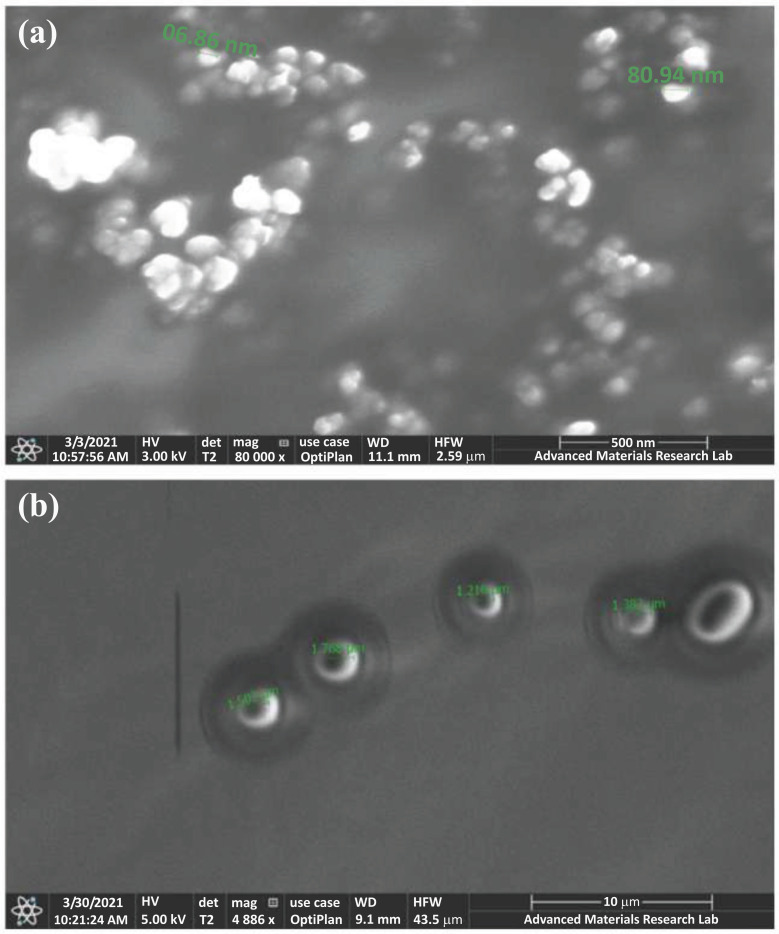
SEM photographs **a**) Blank formula and; **b**) F15 transfersomes. The mean vesicle size of HC-loaded transfersomes significantly increased (*p*< 0.05) than the blank transfersomes, indicating the drug’s loading into the vesicles.

Observations of the SEM photographs showed a smooth surface and spherical structure of the prepared transfersomes for both the blank and loaded formulations. The particle size of F15 was more significant than the blank transfersomes (*p*< 0.05). The increased size of F15 proved the drug loading into the transfersomal vesicles. In addition, the particle size values of the transfersomes by this technique were close to those obtained by the nano-sizer (Fig. **8a**, **b**).

### 3.10 *Ex Vivo* Permeation Study

**Fig. (9):**
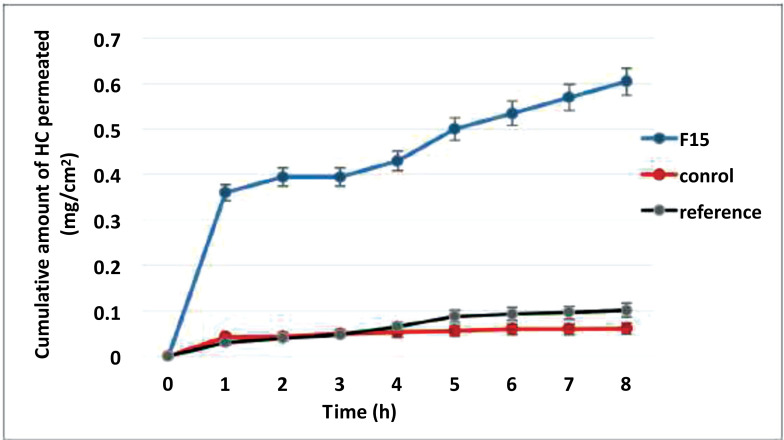
Cumulative HC permeated amount from F15 the reference product and the control through excised rat’s skin. Results are mean± SD (n =3).

The ability of candidate formula F15 to enhance permeation through isolated animal skin was compared to the reference and control (HC in PBS, pH 7.4). Fick’s law was utilized to determine the flux. The permeated amount of HC through the rat’s skin with the selected formula F15 with time is represented in Fig. **9**. 

After 8 hours, the permeated amount from the control and the reference product was lower than that from the transfersomal formulation F15. The enhancement ratio (ER) of F15 was 12.58, whereas the ER of the reference product was 3.89, entailing that the transfersomal formulation enhanced the transdermal delivery of HC significantly (*p*< 0.05), as shown in Table **6**. Previous studies have proposed different mechanisms for the improved transdermal permeation of the transfersomes such as transfersomes containing polar lipids (EA and phospholipids), attracting water *via* the lipid residues-water molecules interaction. Therefore, when transfersomes come in contact with partially dehydrated skin, they move along the hydration medium to escape drying, penetrate the skin, and reach deeper layers. Furthermore, the second mechanism of transfersomes as penetration enhancers is by modifying the intercellular lipids of the stratum corneum, making it weaker, and enabling drugs to penetrate in more significant amounts. Finally, phospholipids, the bone structure of transfersomes, can mix up with the intercellular lipid layers and improve the drug’s permeation [38, 39].

### 3.11 Relation between *In Vitro* Release and *Ex Vivo* Permeation

Linear regression analysis was performed to correlate the percentage of drug release *in vitro* to the percentage of drug permeated in the *ex vivo* study for the selected formula F15. Indeed, such analysis was performed to assess whether HC's *in vitro* release profile from the transfersomes could predict the *in vivo* drug uptake through the skin to reach circulation. However, since the continuous increase of the percentage of HC permeated did not correspond to a relevant increase in the percentage of drug released, where the maximum released amount (≈ 87%) of HC from the vesicles was after 4h and continued approximately constant until the end run. Therefore, the points at time intervals (4, 5, 6, and 7) were excluded from the correlation.

**Fig. (10):**
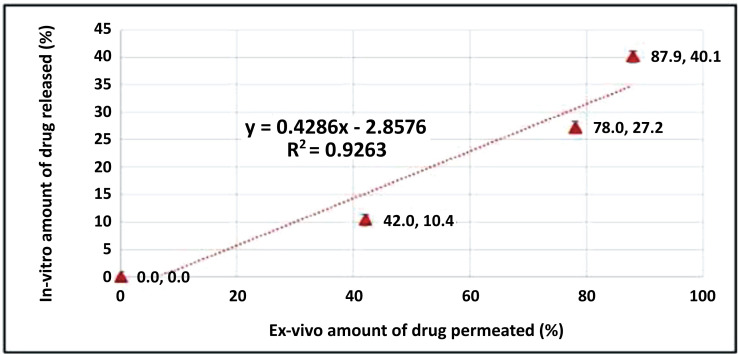
Linear correlation of the percentage of drugs permeated in the *ex vivo* study compared to the percentage of drug release *in vitro* for the selected formula F15. The short Error Bars indicate the values are concentrated and reliable.

The correlation coefficient (R^2^) value was 0.9217, indicating a good linear correlation between the *in vitro* and *in vivo* data of F15 (Fig. **10**). Thus, we expect that the prepared transfersomes would give an improved HC transdermal absorption. However, we believe that *in vivo* clinical studies are essential to prove F15 therapeutic efficiency.

### 3.12 Skin Irritation Assessment

**Fig. (11):**
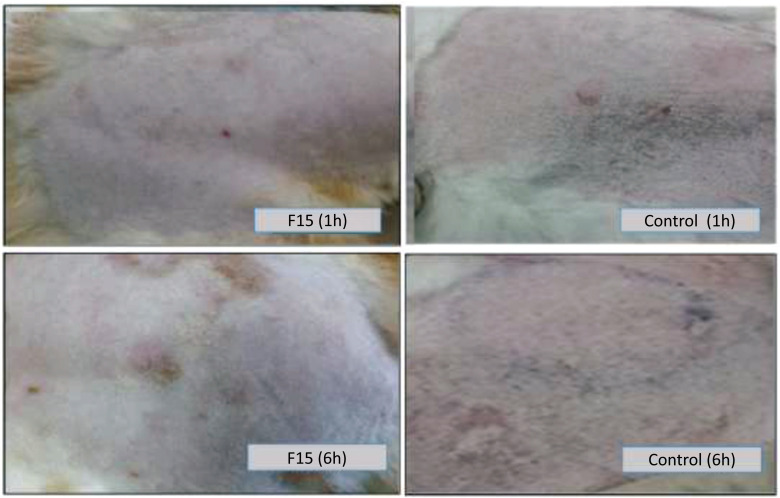
Pictures of the skin irritation study carried out in rabbits for the control (histamine injection) and F15 transfersomal formula; 1h and 6h after sample application.

After injection of the Trypan blue solution, the results showed that the histamine site gradually turned blue, with maximum blue coloration occurring after six hours (Fig. **11**). Thus, the intensity of blue staining at the histamine site served as a reference compared to the other sites. The intensity of the blue coloration increased only at the histamine site. Therefore, the tested HC-Transfersomal formulation, F15, was classified as non-irritant and skin tolerant.

## CONCLUSION

HC was successfully loaded into the transfersomes containing The main constituents of EPC phospholipids include EAs in various ratios and HLBs, such as SC, Span 20, Brij 52, Brij 58, and Span 80. The conditions for preparing the optimized preparation were 12 mL of ethanol as solvent, 9 mL of PBS as hydration medium, and one hour of hydration time. Candidate formula F15 (containing 75 mg Span 80) was selected based on significant (p < 0.05) EE % (69.4±0.80) and optimized *in vitro* drug release (Q8= 87.9±0.62%) compared to other formulations. F15 was characterized by a small mean particle size of 1381±0.20 nm and exhibited satisfactory zeta potential (-8.5±0.11 mV) and elasticity (78.3±1.9 mg s^-1^ cm^-2^). In the quantitative analysis of the *in vitro* drug release data using DDSolver, Weibull modeling was the best kinetic modeling to describe the *in vitro* release data. The n-value of the Korsmeyer-Peppas equation was less than 0.5, indicating a Fickian diffusion mechanism. The simulated pharmacokinetic parameters confirmed that F15 was the preferred formula among the others. Moreover, the HC permeation of F15 through the skin of rats was increased 12-fold compared to the control. The skin irritation test in rabbits proved the safety and skin tolerance of F15 transfersomes.

## Figures and Tables

**Fig. (1):**
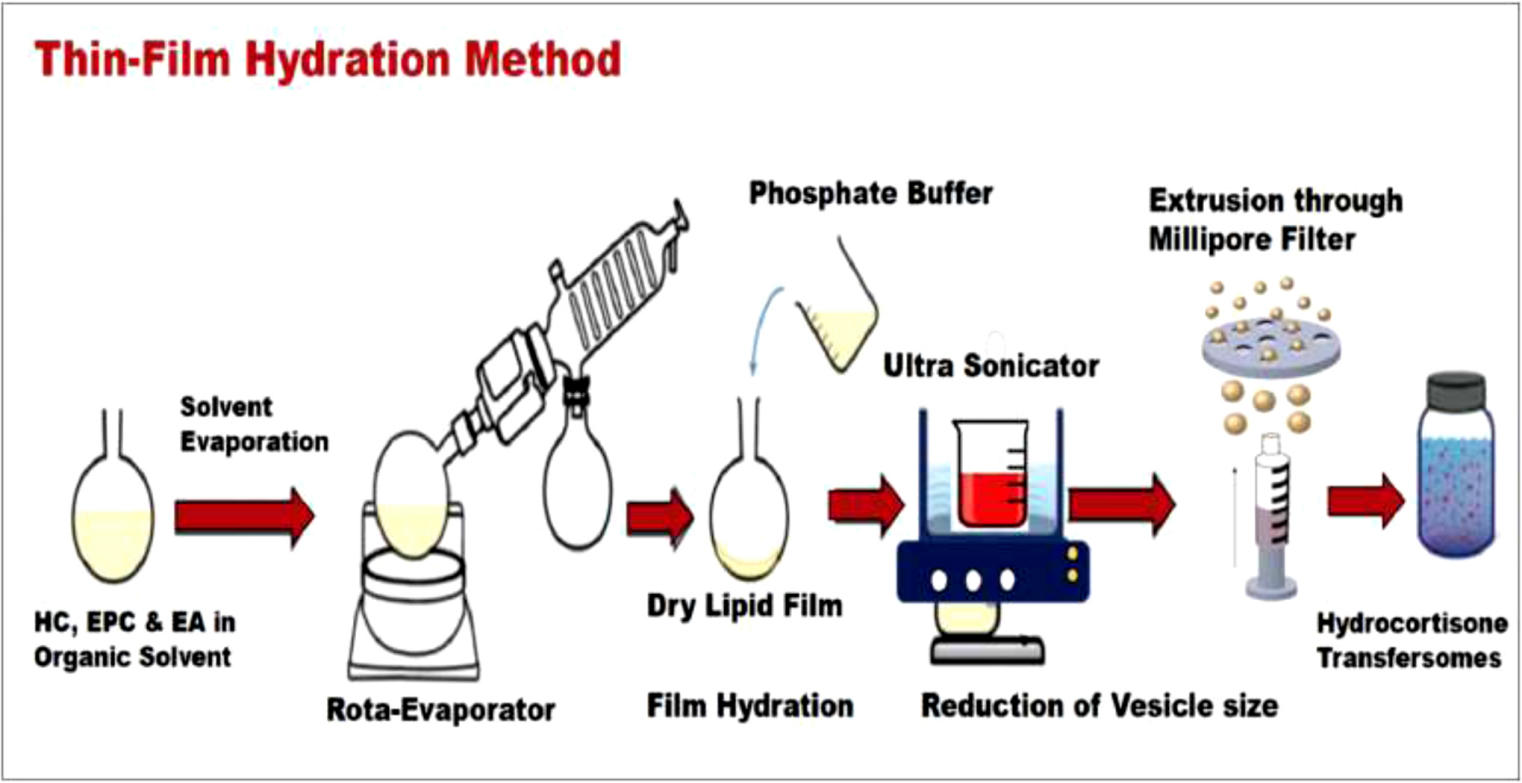
Preparation technique of HC transfersomes by thin-film hydration method.

**Fig. (2):**
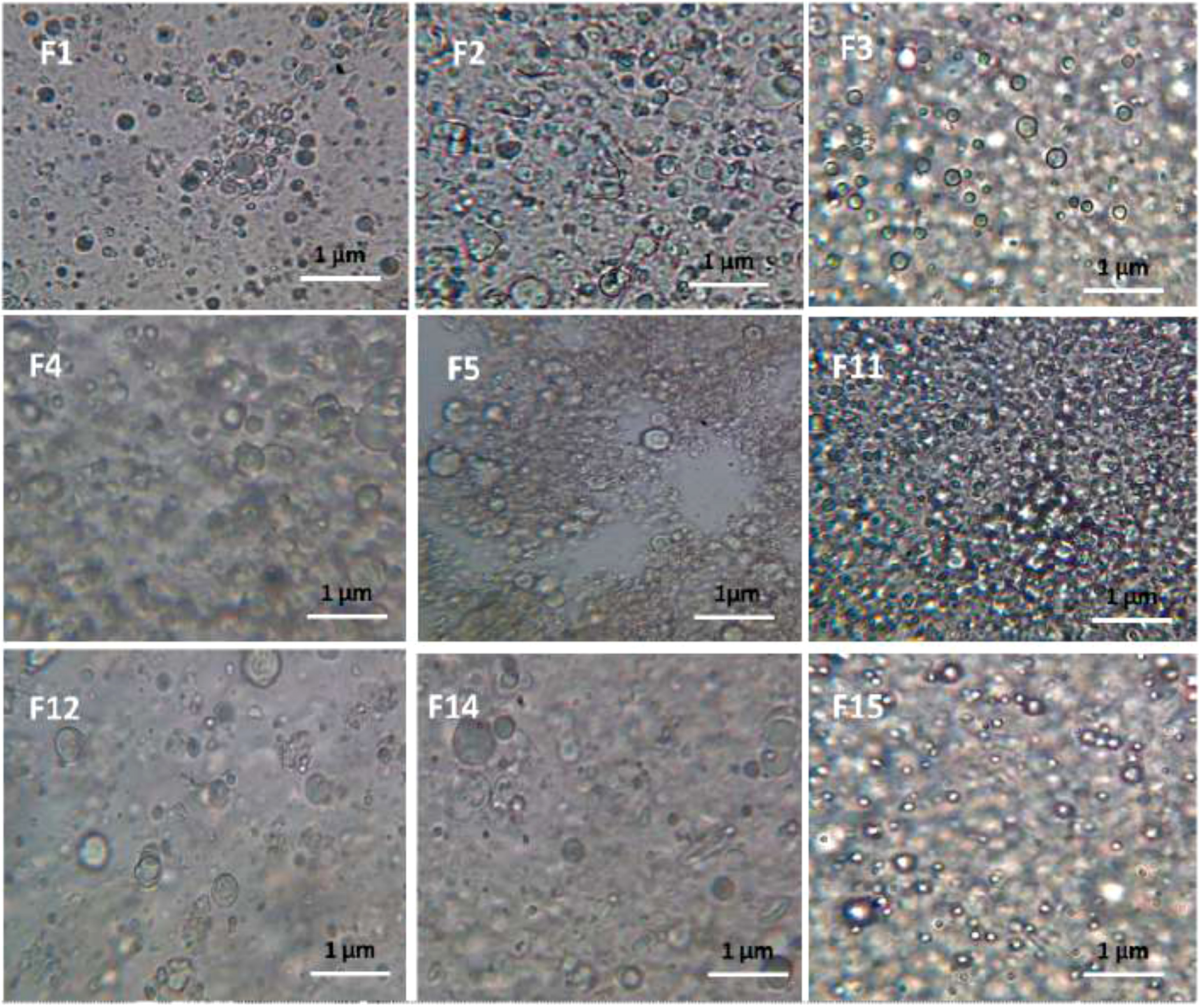
Optical microscopic images of some of the prepared HC transfersomes at 40x magnification.

**Fig. (3):**
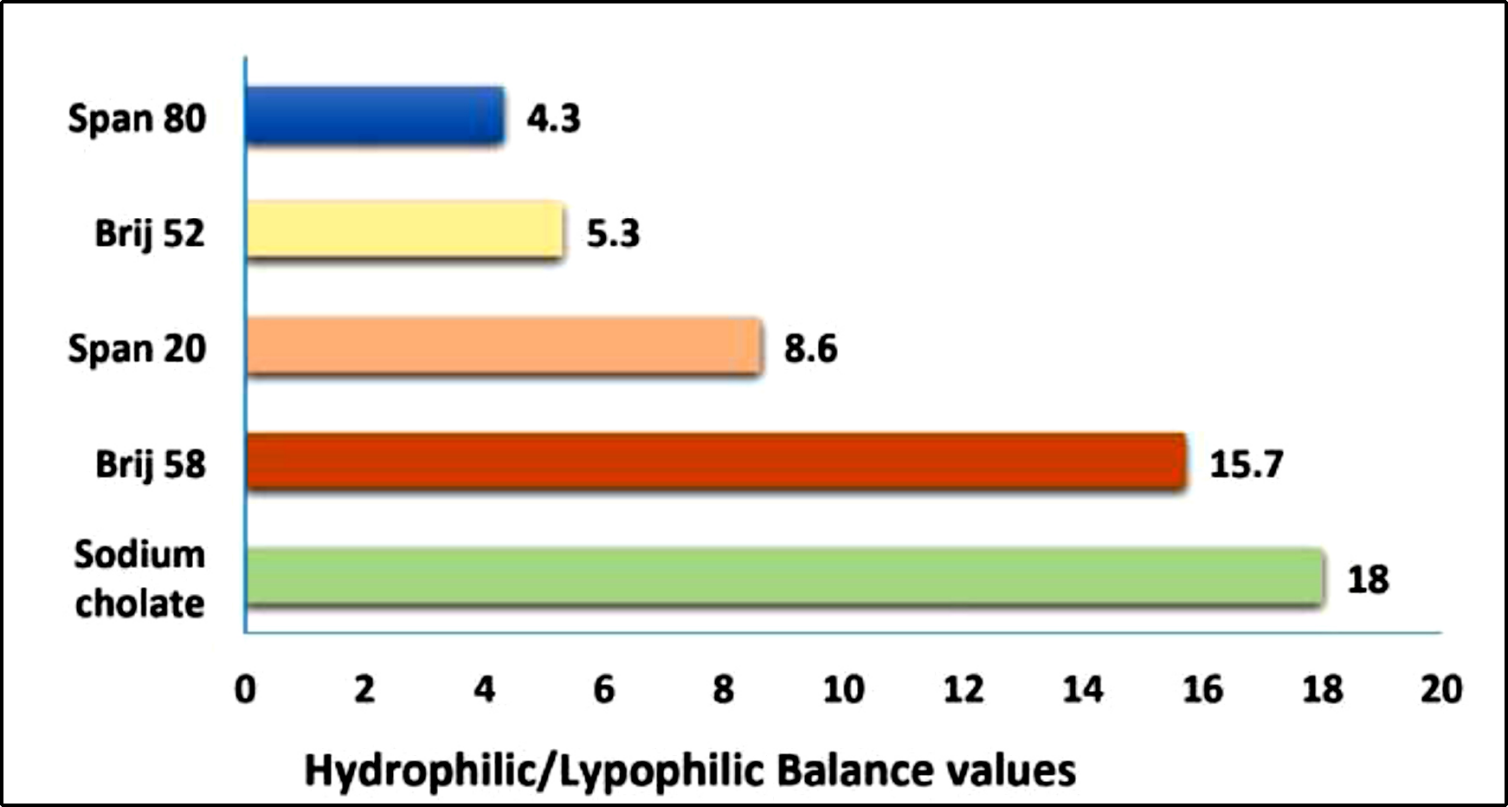
The hydrophilic-hydrophobic balance of edge activators used in HC transfersomes formulations.

**Fig. (4):**
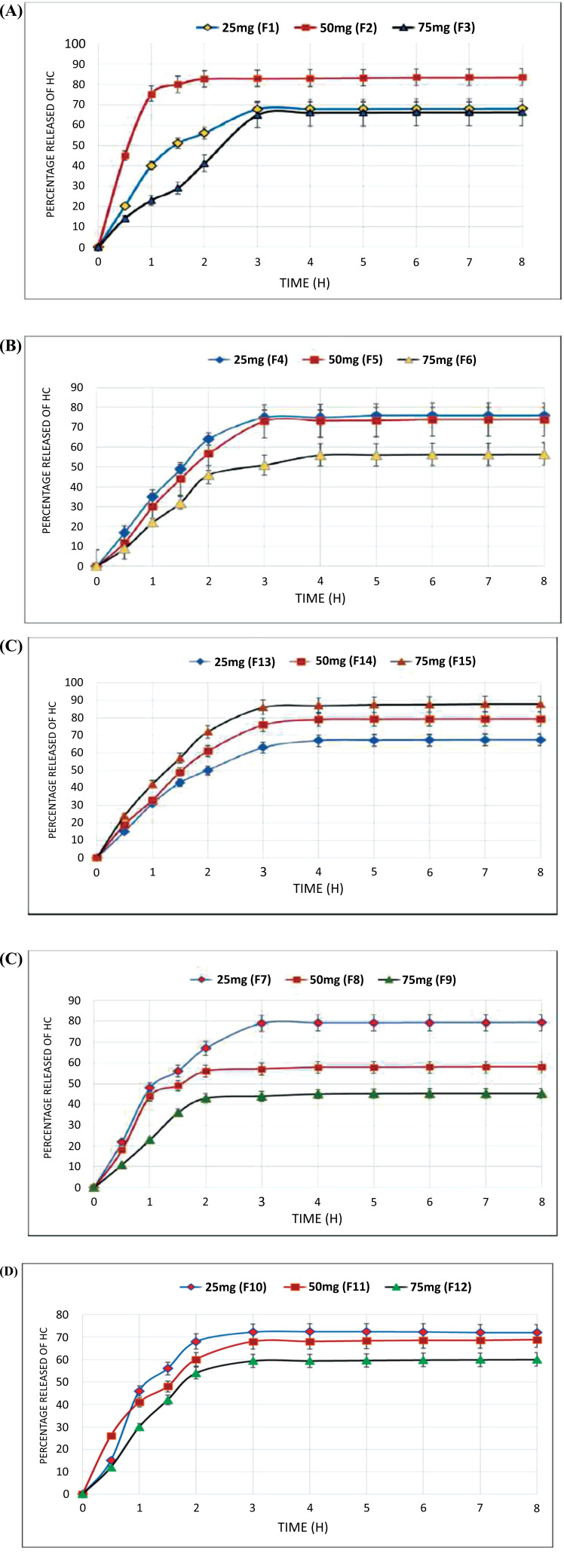
*In vitro* release of HC from the prepared transfersomes containing: **a**) SC; **b**) Span 20; **c**) Span 80; **d**) Brij 52; and **e**) Brij 58.

**Fig. (5):**
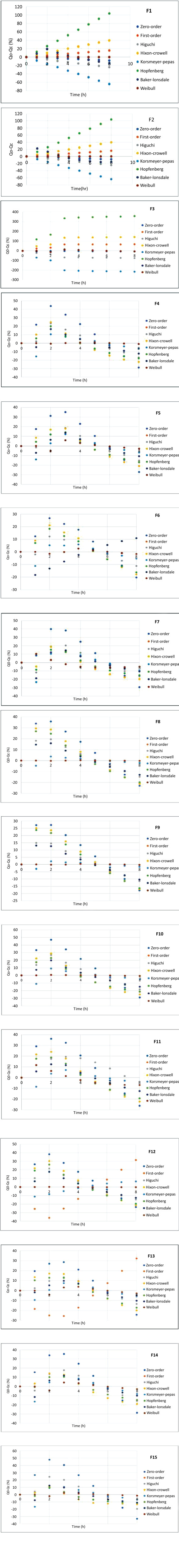
The correlation of residuals (Qo-Qc) *versus* time for HC transfersomal formulations (**F1-F15**) by different dissolution modelings.

**Table 1:** Composition of HC-loaded transfersomal formulations F1-F15.

**Batch #**	**EA**	**EA Amount (mg)**					**EPC:EA (w/w)**
1	SC	25	-	-	-	-	16:1
2	SC	50	-	-	-	-	16:2
3	SC	75	-	-	-	-	16:3
4	Span 20	-	25	-	-	-	16:1
5	Span 20	-	50	-	-	-	16:2
6	Span 20	-	75	-	-	-	16:3
7	Brij 52	-	-	25	-	-	16:1
8	Brij 52	-	-	50	-	-	16:2
9	Brij 52	-	-	75	-	-	16:3
10	Brij 58	-	-	-	25	-	16:1
11	Brij 58	-	-	-	50	-	16:2
12	Brij 58	-	-	-	75	-	16:3
13	Span 80	-	-	-	-	25	16:1
14	Span 80	-	-	-	-	50	16:2
15	Span 80	-	-	-	-	75	16:3

**Table 2:** Kinetic models built in the DDSolver applied to the *in vitro* release data of HC transfersomes.

**Kinetic Models**	**Equations**
**Zero-order**	*Ct=C_0_ +K_0_ t* … … Eq. (2) C is the drug’s initial concentration at time t=0, Ct is the drug amount released at time t, K_0_ is the zero-order rate constant.
**First-order**	*log C=log C_0_-K_1_ t/2.303* … … Eq. (3) C_0_ is the drug’s initial concentration, C is the percentage of the drug’s remaining amount at time t. K_1_ is the first-order rate equation (time^-1^ or per h).
**Higuchi**	*Q=K_H_ × t^1/2^* … … Eq. (4) K_H_ is the Higuchi dissolution constant. Q is the drug amount released at time t.
**Korsmeyer-Peppas**	*Mt/M∞=Kkptn* … … Eq. (5) Mt/M∞ is the drug fraction released at time t, log(Mt/M∞) =log Kkp + nlog t, Kkp is the Korsmeyer release rate constant. Mt is the drug amount released in time t, M∞ is the drug amount released after time ∞ n is the diffusional exponent or drug’s release exponent.
**Hopfenberg**	It describes drug release from infinite cylinders, slabs, and spheres characterized by heterogeneous erosions as: *At/A∞ = 1 – [1-ko t/ Co ao]^n^* … … Eq. (6) At is the drug amount dissolved in time (t); A∞ is the total drug amount dissolved (upon exhaustion of the dosage form; At/A∞ is the drug dissolved fraction; Ko is the erosion rate constant; Co is the initial concentration of drug in the matrix, and ao is the initial radius of the sphere or cylinder or the half-thickness of a slab. The value of ‘n’ is 1, 2, and 3 for a slab, cylinder, and sphere.
**Baker-Lonsdale Model**	It describes the controlled release of the drug from a spherical matrix. *3/2 [1-(1-At/A∞)]-At/A∞ = (3DmCms) / (r0 C0) X t* … … Eq. (7) At is the drug amount released at the time ’t’ A∞ is the amount of drug released at an infinite time, Dm is the diffusion coefficient, Cms is the drug solubility in the matrix, ro is the spherical matrix radius, and Co is the initial concentration of the drug in the matrix.
**Weibull**	*log[–ln(1 – m)] = βlog(t – Ti) – log α* … … Eq. (8) α represents the time scale of the process estimated from X value (t=1), *Ti* portrays the time interval before dissolution starts (*Ti*=0) and β is the shape parameter that characterizes the curve as exponential (β=1), sigmoid (β>1) or parabolic (β<1) and indicates the release mechanism.

**Table 3:** Particle size, PDI, zeta potential, entrapment efficiency, elasticity, and *in vitro* release (Q8) of HC-loaded transfersomal formulations.

**Batch Code**	**EAs**	**Particle Size (nm)**	**PDI** **(%)**	**Zeta Potential (mV)**	**EE** **(%)**	**Elasticity** **(mg s^-1^ cm^-2^)**	**Q8** **(%)**
F1	SC	1302±0.25	27.5±0.01	-6.3±0.10	46.4± 0.72	112± 1.3	68.1±2.05
F2		2045±0.10	30.6±0.06	-8.8±0.05	33.4±0.55	*127± 4.3	83.5 ±1.05
F3		1566±0.20	30.0±0.02	-10.1±0.15	32.9±0.95	133± 2.9	66.4 ±1.36
F4	Span 20	854±0.18	36.2±0.01	-4.6± 0.15	57.1± 0.95	40.1 ± 2.4	76.8±1.52
F5		1489±0.64	35.56±0.01	-4.3 ±0.11	49.1±0.57	44.8 ± 3.9	73.8±1.21
F6		1500±0.63	18.1±0.03	-6.4 ±0.35	41.2±0.57	51.9 ± 1.7	56.4 ± 0.80
F7	Brij 52	1950±0.36	31.3±0.01	-5.0±0.17	42.1±1.15	21.8± 4.5	79.4± 1.28
F8		2092±0.31	32.2±0.02	-5.3±0.10	33.4±1.31	29.4± 2.1	58.4±2.08
F9		2269±0.43	28.7±0.06	-5.2±0.11	57.6±0.76	27.3± 1.1	45.3± 1.69
F10	Brij 58	1315±1.09	31.7±0.03	-5.8±0.68	38.5±0.76	19.3± 1.5	72.2±1.52
F11		1453±0.15	31.4±0.01	-3.5±0.00	32.9±0.55	20.5± 2.1	68.8±0.64
F12		1522±0.15	34.1±0.03	-4.7±0.15	16.1± 0.55	24..8± 1.6	60.2±1.52
F13	Span 80	839±0.21	25.3±0.01	-7.3±0.75	56±0.55	64.3± 2.2	67.4±0.72
F14		1080±0.36	33.1±0.04	-7.1±0.15	57.0±0.64	71.9± 4.1	79.4 ±1.2
F15		1381±0.20	33.2±0.05	-8.5±0.11	*69.4±0.8	78.3± 1.9	*87.9±0.6

Results are given as mean ±SD. * Significant results at *p*< 0.05.

**Table 4:** *In vitro* release kinetics data of HC transfersomal formulations by DDSolver.

**Modeling**	**Formulae**		**Ko**	**R^2^ Adjusted**			**AIC**	**MSC**
**Zero-order**	**F1**		11.87	-0.281			79.423	-2.014
	**F2**		14.65	-0.692			85.127	-2.877
	**F3**		11.13	0.510			72.269	-0.172
	**F4**		13.08	0.083			79.072	-1.246
	**F5**		12.6	0.261			76.868	-0.866
	**F6**		9.58	0.294			71.574	-0.828
	**F7**		13.58	0.311			78.612	-0.628
	**F8**		10.12	-0.421			77.060	-2.408
	**F9**		7.89	-0.420			72.499	-2.440
	**F10**		12.6	-0.271			80.538	-1.974
	**F11**		11.86	-0.074			77.939	-1.692
	**F12**		10.42	-0.225			76.753	-1.887
	**F13**		11.46	0.267			74.738	-0.945
	**F14**		13.51	0.324			77.640	-0.720
	**F15**		15.12	0.082			81.585	-1.277
	**Reference **		3.735	0.923			37.583	1.854
**Modeling**	**Formulae**		**K1**	**R^2 ^Adjusted**			**AIC**	**MSC**
**First-order**	**F1**		0.27	0.491			70.949	-1.072
	**F2**		0.96	0.730			68.438	-1.022
	**F3**		0.21	0.844			62.267	0.939
	**F4**		0.34	0.786			65.955	0.210
	**F5**		0.29	0.838			63.668	0.600
	**F6**		0.16	0.671			64.541	-0.047
	**F7**		0.36	0.861			63.951	1.000
	**F8**		0.18	0.222			71.560	-1.797
**Modeling**	**Formulae**		**K1**	**R^2 ^Adjusted**			**AIC**	**MSC**
	**F9**		0.12	0.034			68.976	-2.048
	**F10**		0.33	0.587			70.377	-0.845
	**F11**		0.26	0.620			68.507	-0.644
	**F12**		0.19	0.390			70.416	-1.183
	**F13**		0.23	0.779			64.146	0.231
	**F14**		0.35	0.891			60.760	1.155
	**F15**		0.57	0.937			57.590	1.388
	**Reference**		0.043	0.959			33.511	2.511
**Modeling**	**Formulae**		**Kh**	**R^2 ^Adjusted**			**AIC**	**MSC**
**Higuchi**	**F1**		30.15	0.625			68.309	-0.779
	**F2**		37.47	0.412			75.512	-1.808
	**F3**		27.4	0.868			60.789	1.103
	**F4**		32.84	0.761			66.778	0.119
	**F5**		31.44	0.830			63.599	0.608
	**F6**		23.86	0.840			57.886	0.692
	**F7**		33.84	0.791			67.521	0.603
	**F8**		25.72	0.576			66.245	-1.206
	**F9**		20.05	0.571			61.614	-1.230
	**F10**		31.96	0.622			69.479	-0.745
	**F11**		29.88	0.738			65.449	-0.304
	**F12**		26.39	0.650			65.431	-0.629
	**F13**		28.58	0.850			60.193	0.670
	**F14**		33.63	0.841			64.341	0.757
	**F15**		37.96	0.777			69.010	0.119
	**Reference**		30.106	0.943			34.680	2.17
**Modeling**	**Formulae**		**Kkp**	**N**	**R^2^ Adjusted**		**AIC**	**MSC**
**Korsmeyer-Peppas**	**F1**		53.9	0.138	0.942		52.349	0.994
	**F2**		77.9	0.039	0.997		30.178	3.228
	**F3**		32.1	0.403	0.862		61.827	0.987
	**F4**		50.5	0.234	0.901		59.857	0.888
	**F5**		44.0	0.292	0.901		59.609	1.051
	**F6**		33.1	0.299	0.921		53.824	1.143
	**F7**		45.2	0.321	0.834		66.649	0.700
	**F8**		48.6	0.102	0.980		39.658	1.747
	**F9**		37.9	0.101	0.986		31.726	2.090
	**F10**		57.0	0.140	0.934		54.602	0.907
	**F11**		49.5	0.186	0.952		50.745	1.329
	**F12**		46.1	0.152	0.935		51.194	0.951
	**F13**		20.4	0.286	0.935		53.682	1.393
	**F14**		45.5	0.314	0.896		61.631	1.058
	**F15**		58.5	0.233	0.912		61.257	0.980
	**Reference**		6.41	0.69	0.970		29	2.8
**Modeling**	**Formulae**		**K_HB_**	**N**	**R^2^ Adjusted**		**AIC**	**MSC**
**Hopfenberg**	**F1**		0	1703.512	0.426		72.955	-1.295
	**F2**		0	6030.329	0.696		70.439	-1.245
	**F3**		0	3255.564	0.818		64.269	0.716
	**F4**		0	5325.278	0.755		67.958	-0.011
	**F5**		0	3262.222	0.806		65.672	0.378
	**F6**		0	2957.188	0.629		66.544	-0.269
	**F7**		0	3948.73	0.846		65.954	0.777
	**F8**		0	2997.533	0.113		73.562	-2.019
	**F9**		0	1356.531	-0.101		70.979	-2.271
	**F10**		0	6572.937	0.527		72.378	-1.067
	**F11**		0	7824.465	0.568		70.508	-0.866
	**F12**		0	4066.529	0.308		72.418	-1.406
	**F13**		0	774.279	0.741		66.162	0.007
	**F14**		0	6659.254	0.881		62.762	0.932
	**F15**		0	2511.394	0.926		59.596	1.165
	**Reference**		0.001	58.583	0.942		35.570	2.077
**Modeling**	**Formulae**		**K_BL_**	**R^2^ adjusted**			**AIC**	**MSC**
**Baker-Lonsdale**	**F1**		0.025	0.781			63.486	-0.243
	**F2**		0.062	0.753			67.765	-0.948
	**F3**		0.019	0.889			59.026	1.299
	**F4**		0.032	0.882			60.613	0.804
	**F5**		0.028	0.91			57.994	1.231
	**F6**		0.013	0.896			54.301	1.090
	**F7**		0.034	0.870			63.572	1.042
	**F8**		0.017	0.707			62.801	-0.824
	**F9**		0.009	0.671			59.292	-0.972
	**F10**		0.030	0.796			63.99	-0.135
	**F11**		0.025	0.856			59.771	0.326
	**F12**		0.018	0.771			61.653	-0.210
	**F13**		0.021	0.924			54.318	1.323
	**F14**		0.034	0.920			58.438	1.413
	**F15**		0.054	0.927			58.779	1.256
	**Reference**		0.001	0.935			35.869	2.044
**Modeling**	**Formulae**	**Parameters**				**R^2^ adjusted**	**AIC**	**MSC**
**Weibull**		**α**	**𝛽**	***T*_i_**		**-**	**-**	
	**F1**	0.909	0.019	1		0.999	7.117	6.019
	**F2**	0.571	0.014	1		1	-27.40	9.625
	**F3**	1.765	0.408	0.867		0.899	59.535	1.242
	**F4**	0.816	0.092	1		0.999	15.170	5.853
	**F5**	1.042	0.213	0.990		0.980	45.629	2.605
	**F6**	1.554	0.154	0.998		0.994	29.827	3.809
	**F7**	0.816	0.163	1		0.991	40.046	3.656
**Modeling**	**Formulae**	**Parameters**				**R^2^ adjusted**	**AIC**	**MSC**
**Weibull**		**α**	**𝛽**	***T*_i_**		**-**	**-**	
	**F8**	1.210	0.028	1		0.999	-3.987	6.596
	**F9**	1.767	0.038	0.999		0.999	-1.267	5.756
	**F10**	0.802	0.02	1		0.999	10.388	5.820
	**F11**	1.021	0.106	0.997		0.994	32.137	3.397
	**F12**	1.129	0.02	1		0.999	-7.781	7.504
	**F13**	1.289	0.228	0.961		0.983	42.144	2.675
	**F14**	0.945	0.255	0.988		0.986	43.54	3.068
	**F15**	0.622	0.169	0.998		0.997	30.157	4.436
	**Reference**	15.684	0.772	-0.001		0.9689	30.673	2.622

**Table 5:** Simulated pharmacokinetic parameters of HC transfersomal formulations F1-F15 and the reference products generated by DDSolver.

**Formulae**	**AUC** **(μg /mL h)**	**MDT** **(h)**	**DE** **(%)**
**F1**	485.3	0.873	0.600
**F2**	490.6	0.627	0.739
**F3**	418.1	1.203	0.522
**F4**	519.0	1.172	0.648
**F5**	490.8	1.349	0.613
**F6**	371.9	1.407	0.464
**F7**	524.7	1.392	0.655
**F8**	417.5	0.802	0.521
**F9**	325.4	0.816	0.406
**F10**	515.3	0.843	0.644
**F11**	408.0	1.071	0.595
**F12**	364.1	0.932	0.530
**F13**	446.6	1.374	0.558
**F14**	522.6	1.418	0.653
**F15**	599.9	1.475	0.749
**Ref**	128.7	1.046	0.160

**Table 6:** *Ex vivo* permeation parameters of F15 and reference product compared to the control through isolated rat’s skin.

**Formula**	**Amount Permeated After 8h (mg/cm^2^)**	**Flux Jss x 10^-3^** **(mg/cm^2^/hr)**	**R^2^**	**P** **(cm/hr)**	**ER**
**F15**	0.605±0.02	5.71±0.01	0.762	0.0545	*12.58±0.08
**Reference**	0.101±0.05	1.26±0.02	0.948	0.0168	3.89±0.09
**Control**	0.061±0.01	0.56±0.01	0.648	0.0043	---

## Data Availability

Not applicable.

## LIST OF ABBREVIATIONS

EAs: Edge Activators
EPC: Egg Phosphatidylcholine
HC: Hydrocortisone
HLB: Hydrophilic-lipophilic balances
